# A Naturally-Occurring Dominant-Negative Inhibitor of Keap1 Competitively against Its Negative Regulation of Nrf2

**DOI:** 10.3390/ijms19082150

**Published:** 2018-07-24

**Authors:** Lu Qiu, Meng Wang, Yuping Zhu, Yuancai Xiang, Yiguo Zhang

**Affiliations:** The Laboratory of Cell Biochemistry and Topogenetic Regulation, College of Bioengineering and Faculty of Sciences, Chongqing University, No. 174 Shazheng Street, Shapingba District, Chongqing 400044, China; qiulu99999@163.com (L.Q.); 20151901005@cqu.edu.cn (M.W.); zhuyupingzhuyu@163.com (Y.Z.); yuancaix@126.com (Y.X.)

**Keywords:** Keap1, Nrf2, Keap1^ΔC^, alternative splicing, redox stress

## Abstract

Transcription factor Nrf2 (nuclear factor-erythroid 2-related factor 2) is a master regulator of antioxidant and/or electrophile response elements (AREs/EpREs)-driven genes involved in homeostasis, detoxification, and adaptation to various stresses. The cytoprotective activity of Nrf2, though being oppositely involved in both cancer prevention and progression, is critically controlled by Keap1 (Kelch-like ECH-associated protein 1), which is an adaptor subunit of Cullin 3-based E3 ubiquitin ligase and also is a key sensor for oxidative and electrophilic stresses. Here, we first report a novel naturally-occurring mutant of Keap1, designated Keap1^ΔC^, which lacks most of its C-terminal Nrf2-interacting domain essential for inhibition of the cap’n’collar (CNC) basic-region leucine zipper (bZIP) factor. This mutant Keap1^ΔC^ is yielded by translation from an alternatively mRNA-spliced variant lacking the fourth and fifth exons, but their coding sequences are retained in the wild-type *Keap1* locus (with no genomic deletions). Although this variant was found primarily in the human highly-metastatic hepatoma (MHCC97H) cells, it was widely expressed at very lower levels in all other cell lines examined. Such Keap1^ΔC^ retains no or less ability to inhibit Nrf2, so that it functions as a dominant-negative competitor of Keap1 against its inhibition of Nrf2 due to its antagonist effect on Keap1-mediated turnover of Nrf2 protein.

## 1. Introduction

The full-length protein of Keap1 was first identified to consist of 624 amino acids (aa) [1]. To date, there are at least 10 alternative splicing transcripts arising from the single *Keap1* gene (that contains six exons and five introns located in the chromosome 19p13.2) in GenBanks by NCBI (Gene ID:9817) and Ensembl (ENSG00000079999). Nevertheless, none of its protein isoforms have been reported so far. In addition, it is important to note that amino acid mutations of Keap1 were found to highly expressed in the human lung, breast and other somatic cancers, and these mutants were also identified leading to the deteriorative activation of Nrf2 [2,3,4,5,6].

The gene encoding Nrf2 was cloned in 1994 and later identified as one of the most important members of the CNC-bZIP family [7,8]. It is generally accepted as a master regulator of AREs/EpREs-driven genes, which adaptive cytoprotection against various stresses [9,10]. In fact, wild-type Keap1 was originally identified to act as a Nrf2-specific inhibitor until 1999 [1,11] and shares highly evolutionary conservation with the *Drosophila* Kelch protein, which is essential for the formation of actin-rich intracellular bridges termed ring canals [12]. Further studies have demonstrated that the negative regulation of Nrf2 by Keap1 is exerted through direct interaction of its C-terminal six double glycine-repeat (DGR)-adjoining domain with the Neh2 region of this CNC-bZIP protein, controlling the latter protein turnover [13,14]. Structural studies have also revealed that only a functional homodimer of Keap1 with each other’s BTB domains is necessary for direct binding to the ETGE and DLG motifs within the Neh2 domain of Nrf2 [14], which acts as a degron targeting the CNC-bZIP protein to ubiquitin-mediated proteasomal degradation pathways. When upon stimulation of Keap1 by oxidative and electrophilic stresses, Nrf2 is dissociated from Keap1-sequestered confinements to be translocated into the nucleus before transactivating AREs/EpREs-battery genes, such as heme oxygenase-1 (HO-1), glutamate-cysteine ligase modifier subunit (GCLM), and NAD(P)H:quinone dehydrogenase 1 (NQO1) [15,16,17].

In the present study, we first report a discovery of a naturally-occurring mutant of Keap1 (designated Keap1^ΔC^), which is expressed primarily in human highly-metastatic hepatoma MHCC97H cells, albeit it was widely expressed at much lower levels in all other cell lines examined. Keap1^ΔC^ is identified to arise from translation of an alternatively mRNA-spliced variant lacking the fourth and fifth exons of wild-type *Keap1* (but with no genomic deletion mutants). The resultant lack leads to a deletion of most of the Keap1 C-terminal domains required for binding Nrf2, to yield a dominant-negative mutant Keap1^ΔC^, acting as a competitor against inhibition of Nrf2-target genes by Keap1.

## 2. Results and Discussion

### 2.1. Discovery of KEAP1^ΔC^ as an Alternatively-Spliced Variant

**Figure 1:**
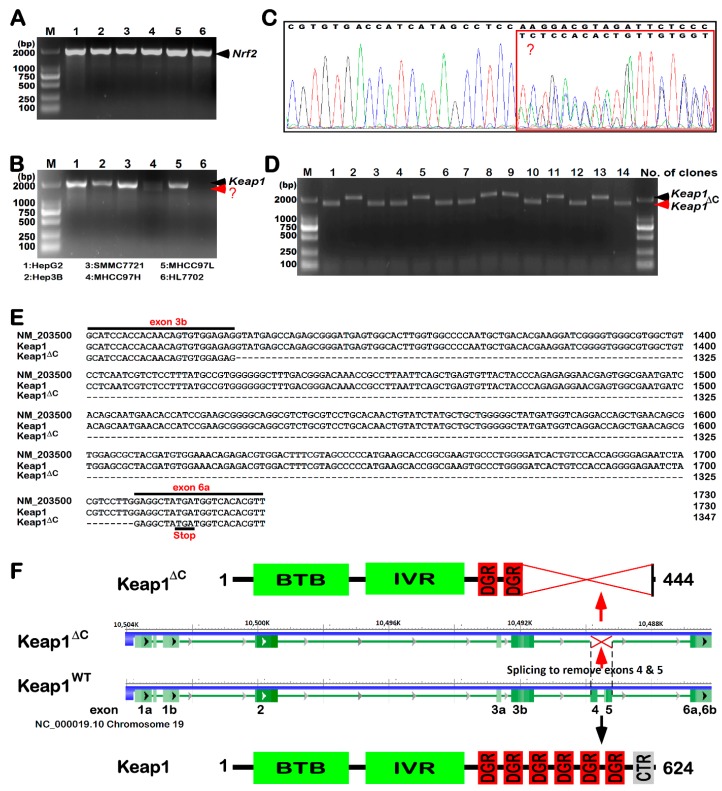
A novel discovery of the *Keap1^ΔC^* mutant. Similar but different PCR (Polymerase Chain Reaction) products of *Nrf2* (**A**) and/or *Keap1* (**B**) were determined by 1% Agarose gel electrophoresis of samples from six cell lines as indicated on the bottom. The red arrow and “?” indicate an unknown band. (**C**) The products of *Keap1* from MHCC97H cells were identified by cDNA sequencing and then analyzed by using the Chromas software. The overlapped peaks of the sequencing curves were placed in a red box. (**D**) Single colonies of *E. coli* bringing a pcDNA3-based expression plasmid (in which the PCR products of *Keap1* from MHCC97H cells were inserted) were subjected to identification of the inserted cDNA fragments by 1% Agarose gel electrophoresis. The black arrow indicates *Keap1* and the red arrow indicates *Keap1^ΔC^*. (**E**) A nucleotide sequence alignment of *Keap1* and *Keap1^ΔC^* was listed, using the DNAMAN software, of which the GenBack Accession No. NM_203500 sequence served as a standard. The black lines means the consensus sequence. (**F**) Shows schematic of distinct lengths of *Keap1* and *Keap1^ΔC^* mRNAs, as well as structural domains of their proteins. Abbreviations: BTB, broad complex, tramtrack and bric-à-brac domain; IVR, intervening region; DGR, double glycine repeat; and CTR, C-terminal region of Keap1.

Since Nrf2 is highly expressed in various cancer cells, to confirm whether there exists a similar potential relationship between Keap1 and Nrf2, as accompanied by their mutants in hepatocarcinogenesis, we determined endogenous expression at their mRNA levels by real-time qPCR (quantitative polymerase chain reaction) analysis of five hepatocellular carcinoma cell lines, followed by sequencing of their cDNAs. As shown in Figure 1A, no changes in mRNA expression of Nrf2 were observed in these cancer cell lines, also with not any mutants examined. By contrast, a considerable lower mRNA expression level of Keap1 was undetectable in HL7702 cells, a non-cancerous hepatocyte cell line, but not in other hepatocellular carcinoma cells (Figure 1B). Interestingly, only the highly-metastatic hepatoma MHCC97H cells gave rise to double cDNA bands of Keap1, at much lower levels than those obtained from all other hepatocellular carcinoma cell lines, which just gave a single band of its full-length. Subsequent sequencing of the PCR products from MHCC97H cells revealed that there overlapped double peaks emerging from the 1326th nucleotide of the Keap1-coding region (Figure 1C).

To clarify the overlapped double peaks of the above-described Keap1 cDNA sequences, they were subcloned into the pcDNA3 expression vector, before 14 clones were randomly selected and identified by PCR with a pair of Keap1 primers as indicated. The resulting electrophoresis of PCR products showed two types of bands with distinct sizes, though both were migrated closely to the standard 2000-bp DNA marker, on 1.0% Agarose gels (Figure 1D). These two bands of Keap1 cDNA were also determined by further sequencing of all selected clones. The result demonstrates that the relatively larger bands represent the wild-type full-length cDNA sequence of Keap1 without any mutation, whilst the shorter bands are owing to a loss of the entire fourth and fifth exons when compared with the intact wild-type (Figure 1E,F). Together with not any of the genomic deletion mutants of the *Keap1* gene locus in these cells examined, these findings have led us to postulate there exists a novel alternatively-spliced variant of Keap1 transcript in MHCC97H cells. Further bioinformatics analysis of the spliced variant revealed a loss of 180-aa residues in the C-terminal of Keap1 (called Keap1^ΔC^, Figure 1F). This results in a constructive deletion mutant of most of the DGR domain and adjacent CTR region of Keap1 that are essential for its interaction with Nrf2 [2,18]. Lastly, the nucleotide sequence of the variant Keap1^ΔC^ along with its amino acid sequence had been submitted to the GenBank developed by NCBI, with the accession No. MG770405.1 being granted.

### 2.2. Differential Expression of Keap1^ΔC^ in Distinct Cell Lines

**Figure 2:**
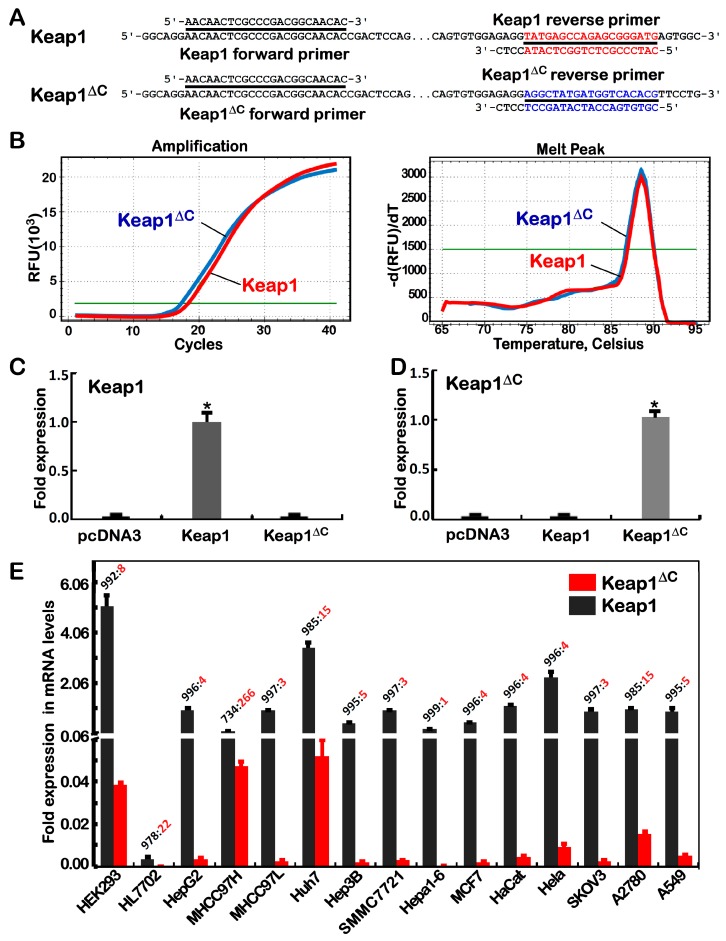
Distinctive expression of *Keap1^ΔC^* in different cell lines. (**A**) Two pairs of primers were designed for specific amplification of *Keap1* and *Keap1^ΔC^*. Their forward primers were identical, but their reverse primers had only four identical bases at the 3′ ends, the different colors means the difference in downstream primers. (**B**) The *Keap1*- and *Keap1^ΔC^*-specific primers were identified by almost overlapped amplification curves (*left*) and melting curves (*right*) of real-time qPCR. (**C**,**D**) Two distinct expression constructs for Keap1 and Keap1^ΔC^ were transfected into HepG2 cells. The total RNAs were extracted and subjected to real-time qPCR to detect distinctive expression of *Keap1*- and *Keap1^ΔC^*-specific mRNAs (* *p* < 0.01, *n* = 3 × 3), respectively. (**E**) Different expression ratios of *Keap1* to *Keap1^ΔC^* at mRNA levels were determined by real-time qPCR analysis of 15 distinct cell lines as indicated.

To determine whether Keap1^ΔC^ is also expressed in other cell lines rather than MHCC97H cells, two distinct pairs of primers targeting specifically for this variant or wild-type *Keap1* (Figure 2A) were, according to their sequence similarity and difference, designed with the same upstream primers coupled with distinct downstream primers retaining the identical four nucleotides (5′-CCTC-3′) at their 3′-ends, for a precision measure of real-time qPCR. To validate the real-time qPCR with distinct pairs of primers, each of expression constructs for Keap1, Keap1^ΔC^ or empty pcDNA3 vector was transfected into HepG2 cells and then subjected to quantitative analysis. Subsequent results illustrated that different products for Keap1 and Keap1^ΔC^ exhibited the same compatible melt peaks (Figure 2B, left), as accompanied by almost overlapped curves of their similar exponential amplification reactions (right panel). As anticipated, the mRNA levels of ectopically-transfected *Keap1* and *Keap1^ΔC^* were only detected by real-time qPCR with their respective specific primer pairs (Figure 2C,D), but not other products of their reciprocal chiasmata were measured.

Further examinations revealed that endogenous *Keap1* and *Keap1^ΔC^* mRNAs were widely but diversely expressed at varying extents in distinct types of 15 human somatic cancerous and non-cancerous cell lines (Figure 2E), including seven hepatoma-derived cell lines HepG2, MHCC97H, MHCC97L, Huh7, Hep3B, SMMC7721 and Hepa1-6, one lung cancer cell line A549, and another four female cancer cell lines MCF7 (from breast cancer), HeLa (from cervical carcinoma), SKOV3 and A2780 (the latter two from ovarian cancer), together with three non-cancerous cell lines of HL7702 (liver), Hacat (skin) and HEK293 (kidney). Overall, expression of *Keap1^ΔC^* mRNA was at relatively higher levels determined primarily in MHCC97H, Huh7 and A2780 cells than other cell lines, but its *bona fide* abundances were also presented at considerably lower levels than the value of wild-type *Keap1* mRNA in all examined cell lines.

### 2.3. Nrf2 Could Be Partially Inhibited by Keap1^ΔC^

**Figure 3:**
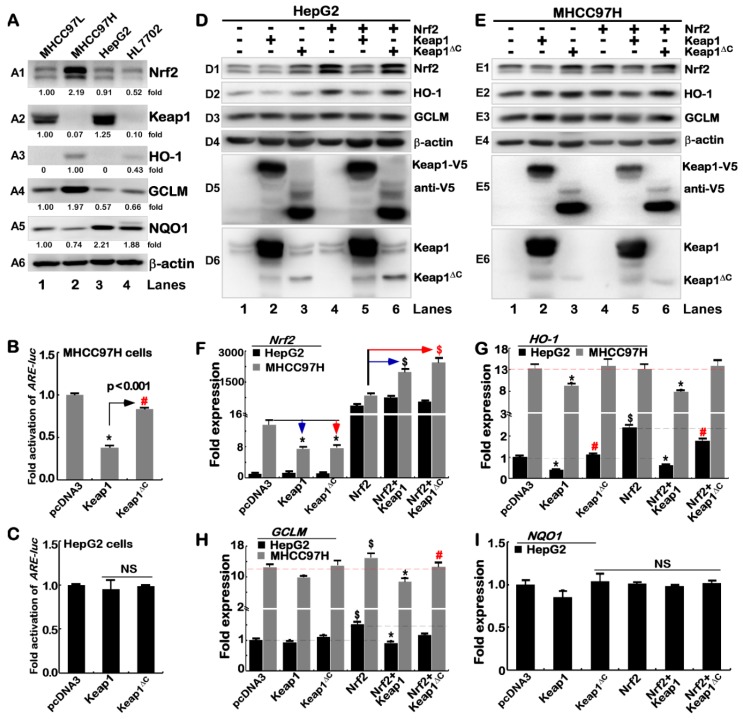
An effect of Keap1^ΔC^ is distinctive from Keap1 on Nrf2-target genes. (**A**) Different expression levels of endogenous proteins Keap1, Nrf2, HO-1, GCLM and NQO1 were determined by Western blotting of four distinct cell lines as indicated. β-actin served as a loading control. (**B**,**C**) An *ARE*-driven fluorescent reporter (pARE-Luc), another internal control pRL-TK, together with an expression construct for Keap1 or Keap1^ΔC^, were co-transfected into MHCC97H (**B**) or HepG2 cells (**C**). After 24 h, the luciferase activity was assayed (^#^ *p* < 0.01, * *p* < 0.001, *n* = 3 × 3; NS = not significant). Both HepG2 (**D**)and MHCC97H (**E**) cell lines were or were not co-transfected with expression plasmids for Keap1 or Keap1^ΔC^ alone or plus Nrf2. After 24 h the total lysates were subjected to Western blotting with distinct antibodies against Nrf2, HO-1, GCLM, β-actin, Keap1 and its V5 tag. (**F**–**I**) Similarly transfected cells as described above were subjected to real-time qPCR analysis of *Nrf2*, *HO-1*, *GCLM* and *NQO1* at the mRNA expression levels. The color arrows indicate comparison with the control group, significant increases (# or $ *p* < 0.01, *n* = 3 × 3) and/or significant decreases (* *p* < 0.01, *n* = 3 × 3) were determined with error bars (S.E.).

To investigate whether inhibition of Nrf2 by Keap1 is interfered by distinct expression of Keap1^ΔC^ in four different cell lines, thus we examined basal protein levels of Keap1 and Nrf2, along with its downstream target genes *HO-1*, *GCLM* and *NQO1*. Western blotting showed a significant high abundance of Nrf2 only in MHCC97H cells, rather than other three cell lines determined (Figure 3(A1)). Along with almost none of the Keap1 protein detected in MHCC97H cells (Figure 3(A2)), the Keap1 mRNA level in MHCC97H cells is also low (Figure 1B), but as accompanied by relative higher levels of Keap1^ΔC^ (Figure 2E). This finding indicates that the increase of Nrf2 should be attributable to low expression of Keap1. Conversely, significant higher abundances of Keap1 in MHCC97L and HepG2 cell lines (Figure 3(A2)), led to marked decreases of Nrf2 to similar lower levels that measured from HL7702 cells (Figure 3(A1)). Further examinations of these cancerous and non-cancerous cells revealed a coupled positive and negative correlation of Nrf2 and Keap1, respectively, with basal protein expression levels of Nrf2-target genes HO-1 and GLCM, but not NQO1 (Figure 3(A3–A5)). Such an exception of NQO1 that was highly expressed in HL7702 and HepG2 cell lines (Figure 3(A5)), though accompanied by lower levels of Nrf2, indicates that it may also be regulated by other CNC-bZIP family members (e.g., Nrf1) and other transcription factors (e.g., AP-1) [19,20].

To determine whether inhibition of Nrf2-mediated gene transcription by Keap1 is interfered by Keap1^ΔC^, both MHCC97H and HepG2 cell lines were co-transfected with the Keap1 or Keap1^ΔC^ expression construct together with an ARE-driven luciferase reporter. The results showed that over-expression of Keap1 caused a significant decrease in *ARE-Luc* reporter activity to ~30% of control levels (Figure 3B, that were measured from transfection of MHCC97H cells with an empty pcDNA3 vector and the reporter). However, over-expression of Keap1^ΔC^ only resulted in slight repression of the reporter gene activity in the same MHCC97H cells (Figure 3B). By contrast, Nrf2-transacting *ARE-Luc* reporter activity was almost unaffected by ectopic Keap1 or Keap1^ΔC^ in HepG2 cells (Figure 3C). This is postulated to be owing to a considerable higher background of endogenous Keap1, so that Nrf2-targeted gene reporter may be not over-regulated by further over-expression of ectopic Keap1 or Keap1^ΔC^ in HepG2 cells.

To further determine the interfering role of Keap1^ΔC^ in the negative regulation of Nrf2 by Keap1, HepG2 and MHCC97H cell lines were transfected with a Keap1 or Keap1^ΔC^ expression construct alone or plus Nrf2 plasmids. As expected, Western blotting showed that endogenous protein levels of Nrf2 were obviously reduced (Figure 3(D1,E1), lanes 2 vs. 1) in both cell lines that had been allowed for further over-expression of ectopic Keap1 (Figure 3(D5,D6,E5,E6)). However, forced expression of ectopic Keap1^ΔC^ led to a significant augment in abundances of endogenous Nrf2 protein (lanes 3 vs. 1). Further examinations also revealed that ectopic Keap1, but not Keap1^ΔC^, led to greater or fewer extents of distinct decreases in total levels of both ectopic and endogenous Nrf2 proteins (Figure 3(D1,E1), cf. lanes 5 & 6 with 4). Moreover, variations in protein expression levels of different ARE-driven genes HO-1 (Figure 3(D2,E2)), but not GCLM (Figure 3(D3,E3)), were correlated positively with changed abundances of Nrf2 *per se* in the presence or absence of ectopic Keap1 or Keap1^ΔC^, implying that HO-1 is an optimal marker of Nrf2-target genes.

Intriguingly, real-time qPCR showed that both endogenous and ectopic mRNA levels of *Nrf2* were almost not influenced by over-expression of either Keap1 or Keap1^ΔC^ in HepG2 cells, but the latter two regulators were required for striking suppression of endogenous *Nrf2*, but were also involved in significant promotion of its ectopic expression in MHCC97H (Figure 3F), suggesting that *Nrf2* mRNA expression and/or its stabilization might be monitored by a proper status of Keap1 or Keap1^ΔC^ in MHCC97H cell lines. The difference in mRNA levels of Nrf2 in these two cell lines may be due to the differences of Keap1 in the genetic background. Importantly, further examinations revealed that both basal and Nrf2-mediated levels of *HO-1* mRNA were markedly repressed by ectopic Keap1, rather than Keap1^ΔC^, in HepG2 cells (Figure 3G). By contrast, MHCC97H cells gave rise to an increased expression background of endogenous *HO-1* mRNA, but it was not further enhanced by Nrf2 over-expression, albeit its expression was obviously reduced by Keap1, but with almost none or less of inhibition by Keap1^ΔC^ (Figure 3G). This implies that transcription of *HO-1* is regulated by Nrf2 in a steady-state system. In addition, basal *GCLM* and *NQO1* mRNAs appeared to be unaffected by either Keap1 or Keap1^ΔC^ (Figure 3H,I), but a marginal increase in Nrf2-induced *GCLM* rather than *NQO1* expression was diminished by Keap1. This was roughly not or less decreased by Keap1^ΔC^ in MHCC97H and HepG2 cells.

### 2.4. Keap1^ΔC^ Acts as a Dominant-Negative Competitor Partially against Intact Keap1

**Figure 4:**
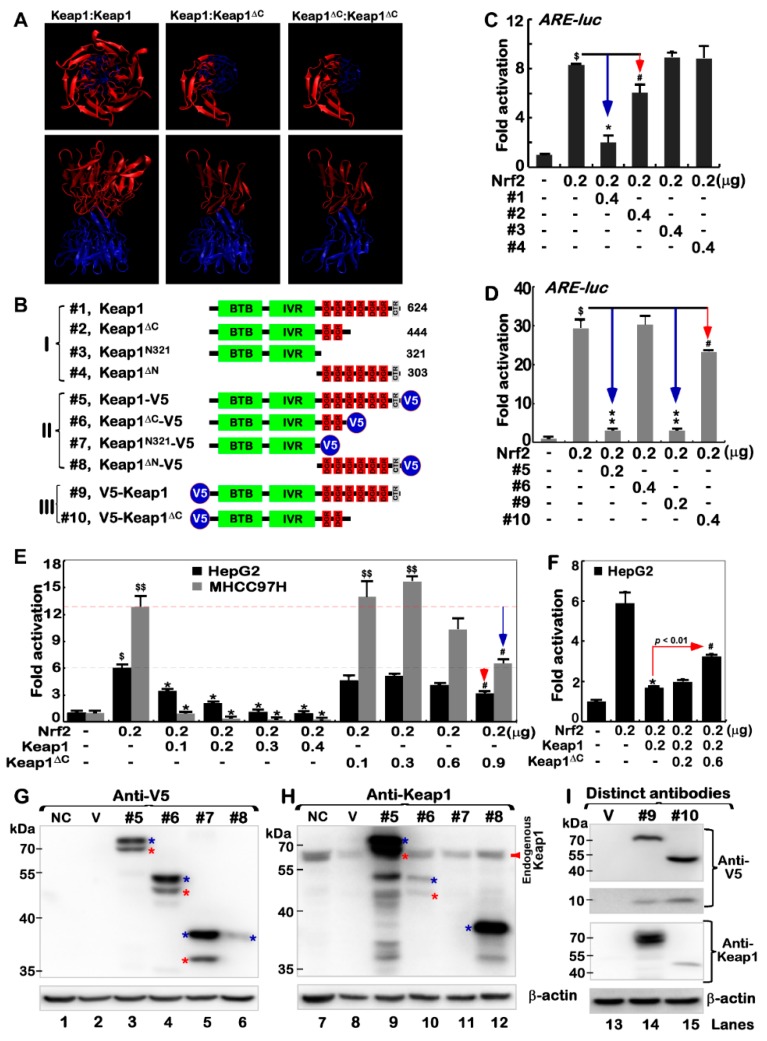
Keap1^ΔC^ competes against effects of Keap1 on the Nrf2-mediated reporter gene. (**A**) Three distinct dimers of Keap1 and Keap1^ΔC^ with different views were simulated by the VMD1.9.3 software, based on a template of the crystal structure of Keap1 (3WN7 deposited in PDB). (**B**) Shows schematic of the structural domains of Keap1, Keap1^ΔC^, as well as other mutants Keap1^N321^ and Keap1^ΔN^, some of which were tagged by the V5 ectope at the N-terminal or C-terminal ends. (**C**,**D**) HepG2 cells were co-transfected with an Nrf2 expression construct, and both reporters of *pARE-Luc* and *pRL-TK* (as an internal control), together with Keap1, Keap1^ΔC^ and other mutants as indicated. After 24 h, luciferase activity was measured with significant changes ($ *p* < 0.001 compared to the normal control group; # *p* < 0.01, * *p* and ** *p* < 0.001 compared to the Nrf2-positive group; *n* = 3 × 3; the color arrows indicate comparison with the control group). (**E**) HepG2 and MHCC97H cell lines that had been co-transfected with a Nrf2 expression construct and two reporters *pARE-Luc* and *pRL-TK*, in combination of distinct cDNA concentrations of Keap1 or Keap1^ΔC^, were subject to luciferase assays to determine ARE-driven reporter activity with significant increases ($ *p* < 0.01; $$ *p* < 0.001 compared to the normal control, *n* = 3 × 3) or significant decreases (*, # *p* < 0.01, compared to the positive Nrf2; *n* = 3 × 3). (**F**) Besides an Nrf2 plasmid and two reporters, *pARE-Luc* and *pRL-TK*, different concentrations of Keap1 and/or Keap1^ΔC^ alone or in combination were co-transfected into HepG2 cells. Then reporter gene activity was measured with significant changes (*n* = 3 × 3; * *p* < 0.001; # *p* < 0.01 compared to the Nrf2 positive group; the color arrows indicate comparison with the control group). (**G**–**I**) Expression constructs for Keap1-V5 (#5), Keap1^ΔC^-V5 (#6), Keap1^N321^-V5 (#7), Keap1^ΔN^-V5 (#8), V5-Keap1 (#9) or V5-Keap1^ΔC^ (#10), along with an empty pcDNA3 vector as the negative control (i.e., V), were transfected into HepG2 cells, respectively. The total lysates of either transfected or untransfected as a normal control (i.e., NC) cells were then analyzed by Western blotting with antibodies against Keap1 and its V5 tag. The blue “*” indicates the larger strip, the red “*” indicates the smaller strip.

To provide a better understanding of the possible reason for that Keap1^ΔC^ is enabled for its interference with Keap1, we conducted molecular modeling of two possible dimeric complexes of Keap1^ΔC^ (only retaining two Ketch motifs) with intact Keap1 (with all six Ketch motifs and adjacent C-terminal region, which are essential for direct association with Nrf2) (Figure 4A). These models were based on a known crystal structure of Keap1 in complex with the N-terminal Neh2 region of Nrf2 (i.e., 3WN7 deposited in the Protein Date Bank) [14,21,22]. From the formation of a possible invalid dimer of Keap1^ΔC^:Keap1 or Keap1^ΔC^:Keap1^ΔC^ (Figure 4A, right panels), it is therefore deduced that they do not only enable intact Keap1 to be simply consumed, but act as a potential dominant-negative competitor of Keap1 against its functional interaction with Nrf2.

To verify whether Keap1^ΔC^ has a marginal negativity to regulate Nrf2 through its residual DGR region, we created a series of expression constructs. Of note, the Keap1^N321^ mutant lacks the entire DGR domain, but retains its N-terminal 321-aa region, while Keap1^ΔN^ is an N-terminal deletion mutant, which comprises only 303 aa covering the entire DGR domain of Keap1 to its C-terminal end (Figure 4B). Subsequently, ARE-driven luciferase reporter assays of HepG2 (Figure 4C) and MHCC97H (Figure 4D) cell lines demonstrated that Nrf2-mediated transactivation activity was significantly suppressed by Keap1, but was only marginally diminished by Keap1^ΔC^. The effect of Keap1^ΔC^ appeared to be influenced by its C-terminal V5 tag (Figure 4D), albeit, it is unknown about its virtual functional folding. Further examinations revealed that Nrf2-target reporter activity was substantially repressed by over-expression of Keap1 in a dose-dependent manner (Figure 4E). However, it is important to note that similar transactivation of this reporter gene mediated by Nrf2 was modestly induced by Keap1^ΔC^ (Figure 4E, right panel). Particularly, inhibition of Nrf2 by Keap1 was blunted by Keap1^ΔC^, leading to a rise in reporter gene activity (Figure 4F). The similar, but different, results were also obtained from an additional aberrant-splicing mutant of Keap1 (with a loss of 458–624 aa) in the human prostate cancer DU-145 cells [23]. Collectively, these findings indicate a possibility that the Keap1^ΔC^ mutant may occupy competitively against intact Keap1 in the formation of an invalid dimer, as a consequence (Figure 4A). Therefore, a putative insufficient interaction of Keap1^ΔC^ with Nrf2 facilitates the CNC-bZIP factor to be released and translocated into the nucleus. In addition, Keap1 is a member of the BTB-BACK-Kelch family and combines with Cul3 to form the E3 ubiquitin ligase complex. However, whether Keap1^ΔC^ can bind Cul3 before functioning as a dominant-negative effect, as the KLHL3 mutant had done [24,25], remains to be further determined.

### 2.5. Putative Processing of Keap1 and Keap1^ΔC^ to Yield Distinct Lengths of Isoforms

The above-mentioned expression constructs (Figure 4B) were also subject to further identification by Western blotting with distinct antibodies against the C-terminal residues 325–624 of Keap1 or the V5 ectopy tagged to the N-terminal or C-terminal ends of this Kelch-like protein (Figure 4G,H). Curiously, the results revealed that Keap1 was likely subjected to the proteolytic processing of the protein within its N-terminal protein by a not-yet-identified protease, in order to yield two distinct lengths of isoforms between ~76 and 70-kDa (both C-terminally tagged by V5). Similar processing of the protein was also examined in additional two cases of Keap1^ΔC^ and Keap1^N321^ (Figure 4G, lanes 3–5). Besides the major ~76-kDa full-length Keap1, its minor isoform of 70-kDa (which was migrated nearly to two close endogenous proteins, Figure 4H) is inferable to be generated after possibly proteolytic removal of a small ~8-kDa N-terminal polypeptide from intact Keap1. This is also further supported by the additional observation that the small N-terminally V5-tagged polypeptide of ~8-kDa was, by coincidence, yielded from the putative N-terminal proteolytic processing of Keap1 or Keap1^ΔC^ (Figure 4I, lanes 14 & 15 in upper two panels). Moreover, immunoblotting of Keap1^ΔN^ unraveled that additional post-synthetic processing of Keap1 might also occur within a region closer to its C-terminal end, such that the majority of the C-terminally-tagged V5 ectopy of this mutant was truncated off the protein, albeit the remaining portion of Keap1^ΔN^ was still recognized by Keap1-specific antibody (Figure 4G, lane 6 vs. Figure 4H, lane 12).

### 2.6. An Antagonist Effect of Keap1^ΔC^ on Keap1-Mediated Turnover of Nrf2

**Figure 5:**
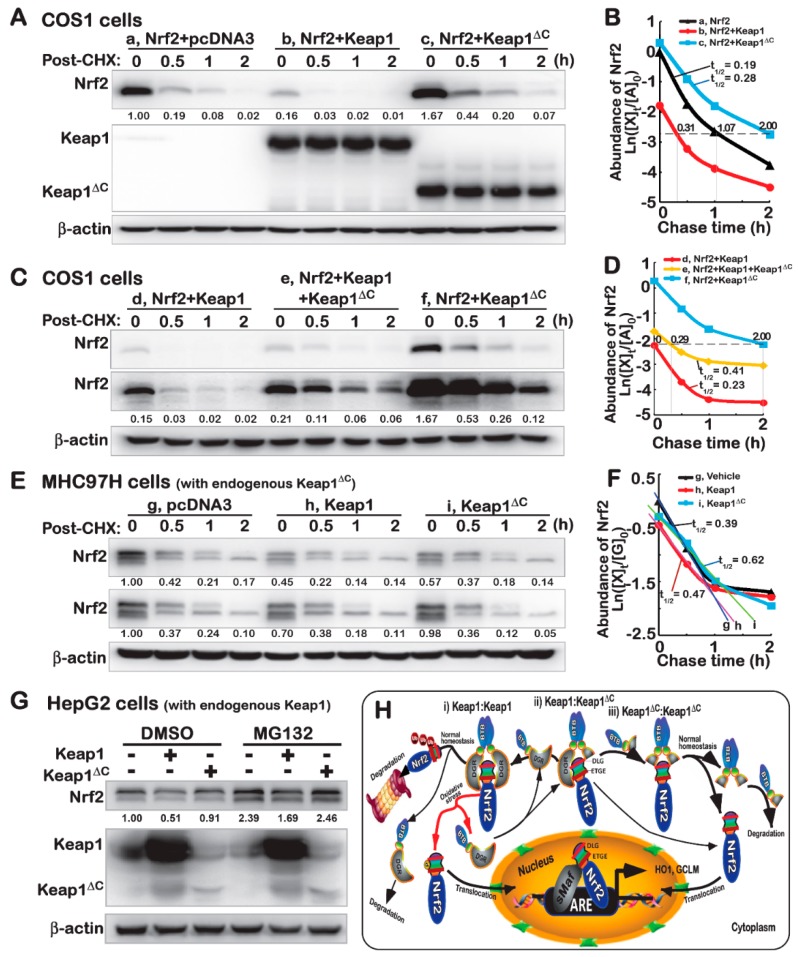
Keap1^ΔC^ has an antagonist effect on the Keap1-mediated turnover of Nrf2. (**A**–**D**) COS-1 cells were transfected with an Nrf2 expression plasmid alone or plus other plasmids V5-Keap1, V5-Keap1^ΔC^ or both, and then treated with 50 μg/mL of cycloheximide (CHX) for distinct indicated lengths of time. The total lysates were determined by Western blotting with antibodies against Nrf2, Keap1, or its V5 tag, respectively. Subsequently, the intensity of blots was quantified and shown on the bottom (**A**,**C**). Of note, relative abundances of Nrf2 and its stability were determined with a changing half-life within distinct setting status, which were calculated and shown graphically (**B**,**D**). (**E**,**F**) MHCC97H and (**G**) HepG2 cells were transfected with an expression construct for V5-Keap1, V5-Keap1^ΔC^ or empty plasmids, before being treated with 50 μg/mL of CHX for indicated times or 10 μMol/L of MG132 for 4 h, prior to being harvested in a lysis buffer. Then, distinct abundances of endogenous Nrf2 proteins were examined by immunoblotting (**E**,**G**). The stability of endogenous Nrf2 protein was also determined with a varying half-life within distinct setting contexts, which was shown graphically (**F**). (**H**) A model is proposed to give a clear explanation of Keap1^ΔC^, acting as a dominant-negative competitor of Keap1. This is due to the fact that Keap1^ΔC^ can occupy the place in the formation of an invalid dimer with Keap1 or itself, no matter whether it only retains less or no ability to inhibit Nrf2. It is important to note that this mutant Keap1^ΔC^ has an antagonist effect on Keap1-mediated turnover of Nrf2 by proteasomal degradation pathway. In addition, upon dissociation of Nrf2 from Keap1, the CNC-bZIP factor will be allowed for spatiotemporal translocation into the nucleus before transactivating ARE-driven cytoprotective genes against oxidative stress or other biological stimuli.

To further explore the potential mechanism by which Keap1^ΔC^ de-represses the negative regulation of Nrf2 by Keap1, distinct settings of pulse-chase experiments were conducted in different cell lines. The COS-1 cells that had been transfected with an Nrf2 expression construct alone or in combination with an additional construct for Keap1 or Keap1^ΔC^, before being treated with cycloheximide (CHX) [26], which inhibits biosynthesis of nascent proteins), were subjected to determination of whether Keap1^ΔC^ exerts an antagonist effect on Keap1-mediated turnover of Nrf2. As anticipated, over-expression of Keap1 caused a strikingly abrupt decrease in the abundance of Nrf2 to 16% of the basal expression levels in the non-Keap1-transfected cells (Figure 5A). Conversely, a substantial increase to 1.67-fold amounts of Nrf2 resulted from co-expression of Keap1^ΔC^. Further examination revealed that over-expression of Keap1^ΔC^ rendered the half-life of Nrf2 turnover to be prolonged from 0.19 h (=11.4 min) to 0.28 h (=16.8 min) following treatment of cells with CHX (Figure 5B). Even after 1-h treatment of cells with CHX, 20% of Nrf2 abundance was retained by Keap1^ΔC^, before being gradually decreased to its putative minimum of ~7% by 2 h of the chemical treatment (Figure 5A). However, just a 7% minimum of Nrf2 was also exhibited at time points of 0.31 h (=18.6 min) or 1.07 h (=64.2 min) respectively, following CHX treatment of cells that had been transfected with Keap1 or not (Figure 5B).

The above-described data demonstrate that Keap1^ΔC^ can extend the half-life of Nrf2, and conversely, may act as an antagonist against endogenous Keap1, leading to disinhibition of Keap1- mediated Nrf2 turnover. To address this hypothesis, COS-1 cells that had been co-transfected with expression constructs for Keap1, Keap1^ΔC^ or both together with Nrf2 plasmids were subjected to further pulse-chase experiments. As the time of CHX treatment was extended to 2 h, a fairly smooth tendency of decreases in the Nrf2 abundance from its maximum (1.67-fold) to its minimum (0.12-fold) was observed in cells co-expressing Keap1^ΔC^ and the CNC-bZIP protein (Figure 5C,D). By sharp contrast, the former minimum of Nrf2 was closer and even almost equal to additional starting level (0.15-fold) of this protein determined immediately before Keap1/Nrf2-co-expressing cells were treated with CHX (Figure 5C, cf. the first lane with the last one). However, the Keap1-led decrease of Nrf2 appeared to be partially mitigated, with a half-life slightly prolonged from 0.23 h to 0.41 h, by co-transfection of Keap1^ΔC^ (Figure 5C,D). This finding indicates that Keap1^ΔC^ exerts an antagonist effect on keap1 in monitoring the turnover of Nrf2 protein.

Further time-course analysis of CHX-treated MHCC97H cells revealed that endogenous Nrf2 protein levels were modestly reduced by over-expression of Keap1, rather than Keap1^ΔC^ (Figure 5E), but its half-life was obviously extended by Keap1^ΔC^ from 0.39 h (=23.4 min) to 0.62 h (=37.2 min) following CHX treatment (Figure 5F). Taken together with the data shown in Figure 2E, these suggest that endogenous Keap1^ΔC^ may compete Keap1, leading to de-repression of Keap1-mediated turnover of Nrf2 protein in MHCC97H cells. However, a marked increase in the endogenous CNC-bZIP protein was observed following treatment of HepG2 cells with proteasomal inhibitor MG132 (at 10 μmol/L) (Figure 5G). Notably, over-expression of Keap1 rather than Keap1^ΔC^ caused significant decreases in baseline abundance and MG132-stimulated accumulation of endogenous Nrf2 protein in HepG2 cells. Collectively, these results demonstrate that Keap1-mediated degradation of Nrf2 in cells is partially inhibited by Keap1^ΔC^ (Figure 5H).

## 3. Materials and Methods

### 3.1. Chemicals, Antibodies and Other Reagents

All chemicals were of the highest quality commercially available. The 26S proteasome inhibitor (MG132, bortezomib) were purchased from Sigma-Aldrich (St. Louis, MO, USA). The primary antibodies such as V5 (Invitrogen, Shanghai, China), KEAP1 (D154142, Sangon Biotech, Shanghai, China), as well as Nrf2 (ab62352) and its target HO1 (ab52947), GCLM (ab126704) and NQO1 (ab80588) (all the latter four antibodies from Abcam, Shanghai, China) were herein employed. In addition to another first antibody against β-actin, various secondary antibodies were obtained from ZSGB-BIO (Beijing, China).

### 3.2. Cell Lines

All these cell lines HEK293, HL7702, HepG2, MHCC97H, MHCC97L, Huh7, Hep3B, SMMC7721, Hepa1-6, MCF7, Hacat, Hela, SKOV3, A2780, and A549 used in this study were purchased from the Cell Bank of Type Culture Collection of Chinese Academy of Sciences (Shanghai, China) and maintained according to the supplier’s instructions.

### 3.3. Expression Constructs and Transfection

The human full-length Nrf2, Keap1 and Keap1^ΔC^ cDNA sequences were subcloned into a pcDNA3 vector, respectively. Deletion mutants of Keap1 were created by inserting appropriate PCR-amplified cDNA fragments into the above-described vector. Related primers were listed in Table 1. These mutants included Keap1^ΔN^ (with a deletion of the N-terminal 321-aa from Keap1), Keap1^N321^ (only retaining the N-terminal 321-aa region). In addition, they were N-terminally or C-terminally tagged by the V5 ectope to yield distinct strategic expression constructs. Following all these constructs were verified by DNA sequencing, they were transfected into experimental cells (2.5 × 10^5^), which had been allowed for growth to a confluence of 80% in 35-mm culture dishes. The transfection was carried out for 15-min in a mixture of indicated constructs (2 μg/mL) with Lipofectamine 3000 (Invitrogen, Carlsbad, CA, USA), and then allowed for a recovery from transfection before being experimented.

### 3.4. Luciferase Reporter Assay

A *pARE-Luc* reporter plasmid [27], which was used for a measure of the transactivation activity of ARE-driven gene mediated by Nrf2, and pRL-TK, which is an internal control of Renilla luciferase reporter, together with indicated expression constructs were co-transfected into HepG2 or MHCC97H cells. At about 24 h after transfection, the luciferase reporter activity was measured by using a dual luciferase reporter assay (Promega, Madison, WI, USA).

### 3.5. Real-Time Quantitative PCR

Experimental cells that had been transfected or not been transfected with the indicated plasmids were subjected to isolation of total RNAs by using the RNAsimple Kit (Tiangen Biotech CO., Beijing, China). Subsequently, 500 ng of total RNAs were added in a reverse-transcriptase reaction to generate the first strand of cDNA (with Revert Aid First Strand Synthesis Kit from Thermo, Shanghai, China). The synthesized cDNA was served as the template for qPCR, in the GoTaq^®^ qPCR Master Mix (from Promega), before being deactivated at 95 °C for 10 min, and amplified by 40 reaction cycles of 15 s at 95 °C and 30 s at 60 °C. The final melting curve was validated to examine the amplification quality, whereas the expression of mRNA for β-actin was viewed as an internal standard control, which is the most stable housekeeping gene selected from multiple housekeeping genes.

### 3.6. Western Blotting

Experimental cells were or were not transfected with indicated constructs for ~8 h, and then allowed for a recovery culture for 24 h in a fresh DMEM containing 25 mmol/L high glucose. The cells were treated with indicated chemicals for 30 min to 4 h before being harvested in denaturing lysis buffer (0.5% SDS, 0.04 mol/L DTT, pH 7.5) that contained the protease inhibitor EASYpacks (1 tablet/10 mL, Roche, Shanghai, China). The lysates were denatured immediately at 100 °C for 10 min, sonicated sufficiently, and diluted in 3× loading buffer (187.5 mmol/L Tris-HCl, pH 6.8, 6% SDS, 30% Glycerol, 150 mmol/L DTT, 0.3% Bromophenol Blue) at 100 °C for 5 min. Subsequently, equal amounts of protein extracts were subjected to separation by SDS-PAGE containing 4–15% polyacrylamide, and visualization by Western blotting with distinct antibodies. On some occasions, the blotted membranes were stripped for 30 min and then re-probed with additional primary antibodies. β-actin served as an internal control to verify equal loading of proteins in each of electrophoretic wells.

### 3.7. Pulse-Chase Experiments

Experimental cells were treated with cycloheximide (CHX, 50 μg/mL) (to inhibit the de novo synthesis of nascent proteins) alone or plus the proteasomal inhibitor MG132 (10 μmol/L), for distinct lengths of time before being harvested. Then, equal amounts of proteins in total cell lysates were subjected to examination of Nrf2 turnover by Western blotting. The intensity of anti-Nrf2 immunoblots was quantified by the Quantity-One software (version: 4.5.2, Hercules, CA, USA) and shown graphically. The half-life of Nrf2 protein was calculated here.

### 3.8. Statistical Analysis

Significant differences in the ARE-driven reporter activity mediated by Nrf2 and its target gene expression levels were statistically determined using either the Student’s *t*-test or Multiple Analysis of Variations (MANOVA). The data are shown as a fold change (mean ± S.D.), each of which represents at least 3 independent experiments that were each performed in triplicate.

## 4. Conclusions

As the most important inhibitory regulator of Nrf2, Keap1 plays a key role in monitoring the protein degradation of this CNC-bZIP factor and its subcellular locations, before regulating ARE-driven target genes [28,29]. Here, we report a novel discovery of Keap1^ΔC^, as a naturally-occurring mutant of Keap1 with a constructive deletion of its C-terminal 180-aa residues essential for its direct binding to the Neh2 domain of Nrf2. The Keap1^ΔC^ mutant is generated from *de novo* translation of the alternatively *Keap1* mRNA-spliced variant lacking both the fourth and fifth exons. The variant Keap1^ΔC^ was determined primarily in the human highly-metastatic hepatoma MHCC97H cells and is widely expressed at very lower levels in all other cell lines examined. Further determination of Keap1^ΔC^ reveals that this mutant protein has a potential at forming an invalid dimer with Keap1 or itself, and it only retains less ability to inhibit Nrf2. On the other hand, the Keap1^ΔC^ is also identified to act as a dominant-negative competitor of Keap1 against its negative regulation of Nrf2, even it was relatively weak. In addition, a line of new evidence has also been provided here, revealing that a putative N-terminal portion of Keap1 (and Keap1^ΔC^) can be proteolytically truncated to yield short isoforms, but this remains to be further determined.

## Figures and Tables

**Table 1:** Distinct paired primers used for expression constructs and RT-qPCR analysis.

Name	Forward (5′-3′)	Reverse (5′-3′)
**1, The following primers used for expression plasmids**		
Keap1	GCTTATCTTCTGGTACCCCATGCA	CAAGAAACTCGAGTTAACAGGTACAGTTCTGCTG
Keap1^ΔC^	GCTTATCTTCTGGTACCCCATGCA	ACTCATCCTCGAGTTATAGCCTCCTCTCCACACTG
Keap1^N321^	GCTTATCTTCTGGTACCCCATGCA	GCGGCCCTCGAGTTACGCCCGGCAGGGCA
Keap1^ΔN^	GATGCCCGGTACCATGCCCAAGGTGGGCCG	CAAGAAACTCGAGTTAACAGGTACAGTTCTGCTG
Keap1:V5	GCTTATCTTCTGGTACCCCATGCA	CAAGAAACTCGAGCGACAGGTACAGTTCTGCTG
Keap1^ΔC^:V5	GCTTATCTTCTGGTACCCCATGCA	ACTCATCCTCGAGCGTAGCCTCCTCTCCACACTG
Keap1^N321^:V5	GCTTATCTTCTGGTACCCCATGCA	GCGGCCCTCGAGCGCGCCCGGCAGGGCA
Keap1^ΔN^:V5	GATGCCCGGTACCATGCCCAAGGTGGGCCG	CAAGAAACTCGAGCGACAGGTACAGTTCTGCTG
Nrf2	GAGCCCGAATTCACGGTCCACAGCTC	AAAACTAGCTCGAGAAAGGTCAAATCCTCCT
V5 tag	CTAGCATGGGTAAGCCTATCCCTAACCCTCTCCTCGGTCTCGATTCTACGGA	AGCTTCCGTAGAATCGAGACCGAGGAGAGGGTTAGGGATAGGCTTACCCATG
**2, The following primers used for RT-qPCR analysis**		
*HO1*	CAGAGCCTGGAAGACACCCTAA	AAACCACCCCAACCCTGCTAT
*GCLM*	GTGTGATGCCACCAGATTTGAC	CACAATGACCGAATACCGCAGT
*NQO1*	AAGAAGAAAGGATGGGAGGTGG	GAACAGACTCGGCAGGATACTGA
*Nrf2*	TCAGCGACGGAAAGAGTATGA	CCACTGGTTTCTGACTGGATGT
*Keap1*	AACAACTCGCCCGACGGCAACAC	CATCCCGCTCTGGCTCATACCTC
*Keap1^ΔC^*	AACAACTCGCCCGACGGCAACAC	CGTGTGACCATCATAGCCTCCTC
*β-actin*	CATGTACGTTGCTATCCAGGC	CTCCTTAATGTCACGCACGAT
